# Experimental and Numerical Investigation on the Strain Response of Distributed Optical Fiber Sensors Bonded to Concrete: Influence of the Adhesive Stiffness on Crack Monitoring Performance

**DOI:** 10.3390/s20185144

**Published:** 2020-09-09

**Authors:** Ismail Alj, Marc Quiertant, Aghiad Khadour, Quentin Grando, Benjamin Terrade, Jean-Claude Renaud, Karim Benzarti

**Affiliations:** 1 MAST, EMGCU, Gustave Eiffel University, IFSTTAR, F-77447 Marne-la-Vallée, France; marc.quiertant@univ-eiffel.fr (M.Q.); benjamin.terrade@univ-eiffel.fr (B.T.); jean-claude.renaud@univ-eiffel.fr (J.-C.R.); 2 COSYS, LISIS, Gustave Eiffel University, IFSTTAR, F-77447 Marne-la-Vallée, France; aghiad.khadour@univ-eiffel.fr; 3 PSN-RES, SEREX, Institut de Radioprotection et de Sûreté Nucléaire (IRSN), CEDEX 13115 Saint-Paul-Lez-Durance, France; quentin.grando@irsn.fr; 4 Laboratoire Navier, Gustave Eiffel University, F-77447 Marne-la-Vallée, France; karim.benzarti@univ-eiffel.fr

**Keywords:** distributed optical fiber sensor (DOFS), strain measurement, polymer adhesive, Young’s modulus, finite element modelling, crack opening

## Abstract

The present study investigated the strain response of a distributed optical fiber sensor (DOFS) sealed in a groove at the surface of a concrete structure using a polymer adhesive and aimed to identify optimal conditions for crack monitoring. A finite element model (FEM) was first proposed to describe the strain transfer process between the host structure and the DOFS core, highlighting the influence of the adhesive stiffness. In a second part, mechanical tests were conducted on concrete specimens instrumented with DOFS bonded/sealed using several adhesives exhibiting a broad stiffness range. Distributed strain profiles were then collected with an interrogation unit based on Rayleigh backscattering. These experiments showed that strain measurements provided by DOFS were consistent with those from conventional sensors and confirmed that bonding DOFS to the concrete structure using soft adhesives allowed to mitigate the amplitude of local strain peaks induced by crack openings, which may prevent the sensor from early breakage. Finally, the FEM was generalized to describe the strain response of bonded DOFS in the presence of crack and an analytical expression relating DOFS peak strain to the crack opening was proposed, which is valid in the domain of elastic behavior of materials and interfaces.

## 1. Introduction

Structural health monitoring (SHM) is a crucial approach in the framework of maintenance and safety management plans of civil structures and nuclear power plants and still remains an active field of research and development. Optical fiber (OF) sensor systems are now considered as one of the most promising technologies for the monitoring of civil engineering structures as this technology is capable of measuring various types of measurands such as pH, temperature, moisture, corrosion, strain, cracks, and parameters related to the chemical composition of the host medium [1,2,3,4,5,6]. However, before selecting an instrumentation for SHM, both the performance of the measurement chain (including the sensing element, waveguide, and interrogation device), the reliability of measurements and the durability of the sensor must be evaluated [5,7,8,9]. In addition, in the particular case of reinforced concrete (RC) structures, the efficiency of the sensing system to detect and localize cracks and to assess crack openings is an important issue for practitioners and infrastructure owners who need to establish real-time diagnosis on the state of wear and damage of the monitored structures [10,11,12]. In this context, SHM instrumentation based on distributed optical fiber sensors (DOFS) enables the monitoring of crack development in concrete at an early stage, before this phenomenon becomes visible to the naked eye [13]. Crack initiation and development lead indeed to the appearance and growth of strain peaks on the DOFS signal, and the peak locations are directly correlated to the positions of cracks in the structure [1,14,15,16]. However, crack opening also induces a high stress concentration over a short length of the DOFS [17], which may lead to premature failure of the sensing element. In this case, strain measurements would be lost at the very beginning of the crack development. For this reason, bare OFs (with their primary coating), which are very fragile, are generally not used directly in the field for long-term operations, except when bonded to the reinforcing bars of RC structures [18,19].

In practice, most commercial products include additional coating layers that protect the core of the OF against mechanical damage and chemical attacks and prevent the sensor from early breakage [15,20,21]. Although these sensing cables are more robust, strain measured by the core fiber may be different from the actual strain in the host structure, due to shear deformation of the coating of the cable. To overcome this bias and produce quantitative strain measurements, a general methodology was introduced in a previous work [22] and has proven to be efficient for both DOFS cables embedded in concrete [21,23] and cables bonded to the surface of existing concrete structures [24,25]. This methodology is based on a modelling of the strain transfer mechanism through the coating of the cable (and also through the adhesive layer in the case of bonded cables), and allows to determine a mechanical transfer function (MTF) that relates the measured strain to the actual strain in the host structure. However, its implementation requires a precise knowledge of the mechanical properties of the various material layers constituting the coating and the determination of all interface behaviors, which can be difficult to obtain from simple experiments, especially for cables showing complex internal structure. In addition, the presence of the protective coating tends to increase the anchorage length of the cable (also called effective length or development length); hence, reducing its sensitivity with regard to crack detection. In this context, designing an external coating that ensures both an appropriate protection of the OF core and a limited impact on crack monitoring performance of the DOFS cable can be very challenging.

The present paper explores another solution aiming to ensure both sensitivity and robustness requirements for DOFS instrumentation applied to the SHM of existing RC structures. Such an approach consists in:

- using bare OF sensors (including their primary coating) that are bonded to the concrete surface with a polymer adhesive; and
- optimizing the mechanical properties of this intermediate adhesive layer in order to mitigate local stress concentrations along the DOFS arising from crack development in concrete, while maintaining a good level of performance for crack detection. In this configuration, strain from the host structure is transferred through the adhesive layer only, which deforms mainly under shear stress [26]. Consequently, the measurement sensitivity of the bonded sensor depends strongly on the characteristics of this adhesive layer.

This type of approach, previously applied to the monitoring of composites structures [27], was rarely investigated in the case of RC structures. For example, Glisic et al. [28] inserted the OF within a tape and selected carefully the adhesive used to bond the sensor in order to allow a partial delamination of the sensing tape. As crack developed in the loaded structure, partial debonding of the tape was reported right above the cracked zone, and the peak stress was then redistributed over the entire detached length, protecting the sensing element from an early failure. 

Barrias et al. [2] evaluated the performance of different adhesives (epoxy, silicone, polyester, and cyanoacrylate) for the bonding of DOFS on the bottom surface of RC beams subjected to three-point bending tests. Under both static and fatigue loads, epoxy showed the best performance and strain values obtained matched perfectly with those from the strain gauges. However, after the elastic regime (when the beam was cracked and loaded up to failure), only DOFS bonded using silicone, the adhesive with the lowest shear modulus, provided realistic results [29].

The present work relies on both theoretical and experimental investigations. In a preliminary part, a simple finite element model (FEM) is proposed to describe the strain transfer process for a specific configuration of surface mounted DOFS, in which the bare OF is bonded/sealed with a polymer adhesive into a groove engraved at the surface of concrete. This FEM is then used to evaluate the sensitivity of the strain response of the sensor to changes in adhesive stiffness, and results are also compared with those derived from simple analytical models from the literature.

In a second part of the study, results of two mechanical tests are presented, i.e., a compression test on a concrete cylinder and a three-point bending test on a notched concrete prism. Both specimens are instrumented with bare DOFS sealed into grooves at the concrete surface using different types of adhesives and interrogated with an optical backscatter reflectometer (OBR) unit based on Rayleigh scattering. Such experiments aim to confirm the influence of the adhesive properties on the strain response and crack detection ability of the bonded DOFS, in the case of multiple crack development or in presence of a single notch-induced crack, respectively.

In the last part of the paper, the FEM approach is extended to describe the strain response of the bonded DOFS when a crack occurs in concrete and crosses the sensor path. An objective is to establish a generalized analytical model that relates the characteristics of the measured strain peak to the crack width, based on the actual geometrical configuration of the DOFS instrumentation.

## 2. Theoretical Analysis of the Strain Transfer Process between Concrete/Bonded DOFS

### 2.1. Representative Model Configuration

In the present study, practical installation of bonded DOFS on a concrete specimen requires preliminary carving of a 2 mm wide groove at the concrete surface using a concrete saw. A regular single mode OF (external diameter 160 μm, including the core fiber and its polyimide (PI) coating) is then introduced in the groove and sealed using a polymer adhesive. 

**Figure 1:**
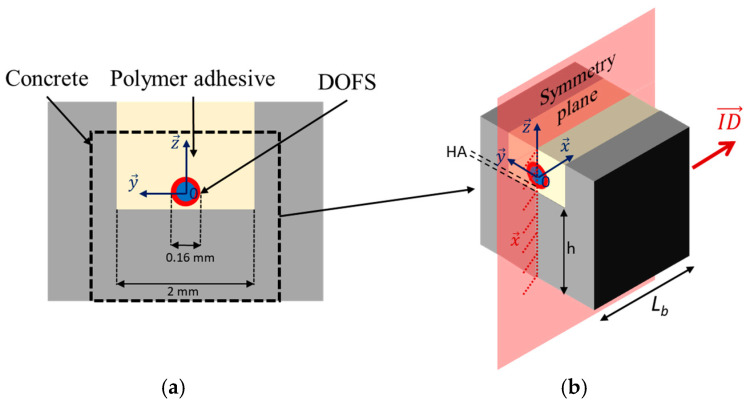
Simplified model used in the numerical simulation of the strain transfer process: (**a**) cross-sectional view of the system; (**b**) 3D model with boundary conditions.

In this context, a simplified 3D FEM was adopted to simulate the strain transfer process between the host concrete substrate and the core fiber (a cross-sectional view of the system is illustrated in Figure 1a). The concrete block of length *L_b_* was subjected to tensile loading by imposing a displacement ($\vec{ID}$) along $\vec{x}$ axis to one side of the block, while displacements along $\vec{x}$ axis were constrained on the opposite side of the block. Due to geometrical symmetry of the system with respect to the (O, $\vec{x},$ $\vec{z}$) plane, only half of the 3D system presented in Figure 1b was modelled.

**Figure 2:**
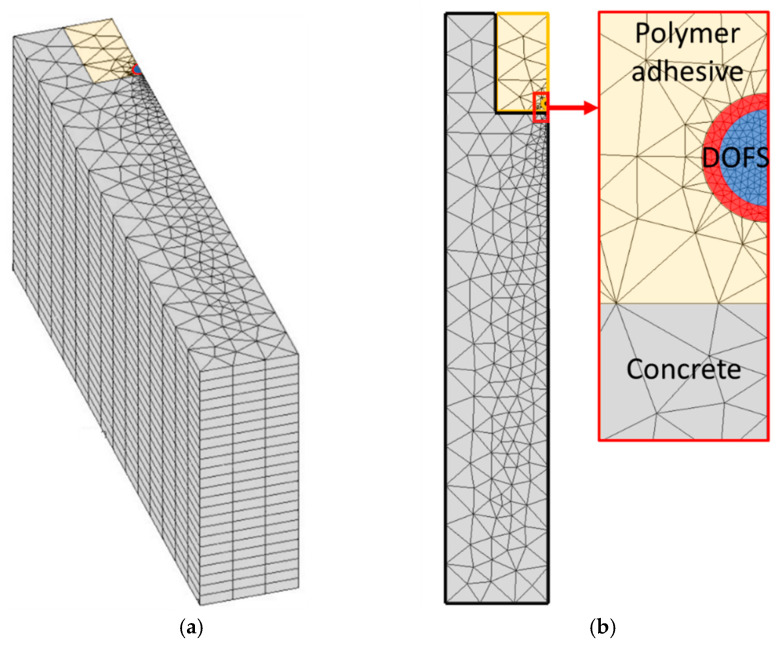
Finite element mesh of (**a**) the 3D geometry and (**b**) the cross-sectional view of the system.

A strain transfer analysis can then be performed following a numerical approach based on this simplified configuration. The 3D FEM was implemented in Cast3m [30], using linear tetrahedral elements. A mesh sensitivity analysis was conducted, and multiple mesh sizes were tested (an example is shown in Figure 2). The number of nodes in the mesh was then optimized to approximately 90,000 in order to minimize both running time and numerical errors. 

A linear elastic mechanical behavior was assumed for all material components and perfect adherence was considered at all interfaces. Values of input parameters included in the FEM are listed in Table 1.

In order to provide comparative data, a complementary analysis was carried out using analytical models from the literature which are presented in the next section.

### 2.2. Analytical Approach

Measuring strain along the DOFS can be carried out with a very high precision. However, when an OF is embedded in a host material, the coating layer absorbs a substantial part of the strain transmitted to the glass core. The analytical approach of the strain transfer analysis consists in integrating mechanical and geometrical properties of the different materials from the host material to the glass core of the DOFS, in order to develop an analytical formula, which relates strain in the DOFS glass core (*ε_f_*) to strain of the host medium (*ε*_0_). Variety of analytical formulas were developed in the literature and validated experimentally for OF sensors embedded in concrete. In the specific case of DOFS bonded to the surface of a host material, a portion of the strain is absorbed by the adhesive layer that mainly deforms under shear stress. Hence, the adhesive thickness and stiffness influence the amount of strain transferred from the host material to the FO sensor. In addition, symmetry of the system with respect to the central axis of the DOFS is no longer a valid assumption in the model. Consequently, the development of analytical formulas describing strain transfer becomes more complicated compared to the embedded DOFS configuration. 

Two analytical models introduced in previous researches are presented below. Results provided by these models are then compared to those obtained from the proposed numerical approach.

**Figure 3:**
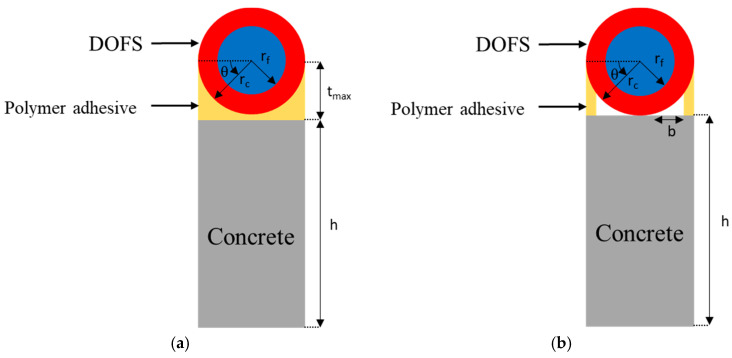
Schemes of the theoretical models developed by (**a**) Kim et al. [31] and (**b**) Her et al. [32].

Kim et al. [31] developed an analytical strain transfer model for DOFS bonded to a concrete surface, based on the scheme illustrated in Figure 3a, using the following hypotheses: all materials exhibit linear elastic behavior, adherence is considered perfect at all interfaces, the adhesive layer is deformed only by shear stress, and the DOFS glass core is deformed only by axial stress.

The strain distribution in the DOFS core is given by the following expression (parameters used in these equations and their meanings are summarized in Table 1):

(1) $$\varepsilon_{f}=\frac{r_{c}\sigma_{0}ConstB}{E_{f}E_{h}\lambda^{2}}\;\int_{0}^{\pi }f\left(\theta \right)d\theta \;\left[1-\frac{cosh\left(\lambda x\right)}{cosh\left(\lambda L_{b}\right)}\right]$$

where

(2) $$\lambda =\sqrt{\frac{r_{c}ConstB.\left(ConstC.E_{f}+E_{h}\right)}{E_{f}E_{h}}\int_{0}^{\pi }f\left(\theta \right)d\theta }$$

(3) $$ConstC=\frac{\pi \left[r_{f}{}^{2}+\frac{E_{c}}{E_{f}}\left(r_{c}{}^{2}-r_{f}{}^{2}\right)\right]}{2hr_{c}}$$

(4) $$\frac{1}{ConstB}=\left\{\pi r_{f}{}^{2}-r_{c}\int_{0}^{\pi }f\left(\theta \right).\frac{E_{c}}{2E_{f}G_{c}}\left[r_{c}{}^{2}\ln\left(\frac{r_{c}}{r_{f}}\right)+\frac{r_{f}{}^{2}-r_{c}{}^{2}}{2}\right]d\theta +\frac{\pi E_{c}}{E_{f}}\left(r_{c}{}^{2}-r_{f}{}^{2}\right)\right\}$$

(5) $$f\left(\theta \right)=\frac{G_{a}G_{c}}{r_{c}G_{a}\ln\left(\frac{r_{c}}{r_{f}}\right)+G_{c}\left(t_{max}-r_{c}sin\theta \right)}$$

(6) $$t\left(\theta \right)=t_{max}-r_{c}sin\theta$$

Another analytical model, proposed by Her et al. [32], assumes the existence of gaps between the DOFS and the host material, which are not filled with polymer adhesive, as illustrated in Figure 3b.

Assumptions of this model are as follows: all materials exhibit linear elastic behavior, all interfaces are perfectly bonded, gaps of width *b* (Equation (8)) are not filled with the polymer adhesive, and the primary coating and the adhesive layer are only subjected to shear stresses.

In these conditions, strain in the DOFS core is given by

(7) $$\varepsilon_{f}=\frac{\varepsilon_{0}}{E_{f}\left(\frac{\pi r_{f}{}^{2}}{2hr_{c}E_{h}}+\frac{1}{E_{f}}\right)}\left[1-\frac{cosh\left(\lambda_{1}x\right)}{cosh\left(\lambda_{1}L_{b}\right)}\right]$$

where

(8) $$\lambda_{1}=\sqrt{\left[\frac{2r_{c}}{\pi r_{f}{}^{2}}\left(\frac{\pi r_{f}{}^{2}}{2hr_{c}E_{h}}+\frac{1}{E_{f}}\right)\int_{0}^{cos^{-1}\left(\frac{b}{r_{c}}\right)}\frac{1}{\frac{r_{c}\left(1-\sin\theta \right)}{G_{a}}+\frac{r_{c}}{G_{c}}\ln\left(\frac{r_{c}}{r_{f}}\right)}d\theta \right]}$$

### 2.3. Comparison between Analytical and Numerical Approaches

It must be highlighted that analytical approaches do not take into account the presence of the groove, unlike the FEM in which the actual experimental configuration is modelled (Figure 1). In order to get comparable results, it is considered in all cases that the bottom of the DOFS is directly in contact with concrete at the bottom of the groove. This means that the height of adhesive layer below the DOFS (noted *HA*) in the following and in Figure 1b is equal to 0 mm in the FEM, $t_{max}\;=\;r_{c\;}$ in Equation (6), and $b\;=\;r_{c}$ in Equation (8).

**Figure 4:**
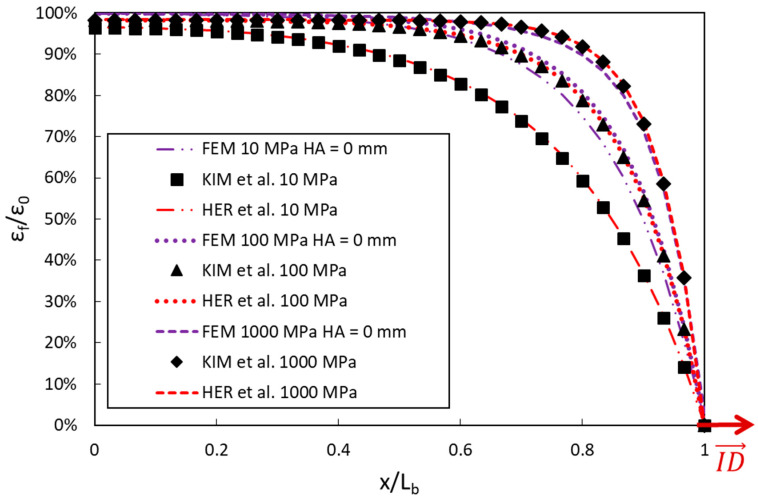
Strain transfer curves provided by the analytical and numerical models, for different values of the adhesive stiffness.

Figure 4 displays the strain transfer curves along the DOFS bonded to the concrete surface, as provided by the two analytical models and the FEM when considering different values of the polymer adhesive stiffness (*E_a_* varying in the range 10–1000 MPa).

From Figure 4, it can be noticed that the two analytical models provide almost the same strain profiles for a given value of the adhesive stiffness. FEM simulations are also very close to those of analytical models as long as the adhesive stiffness is high but reveal significant deviation in the case of softer adhesive (*E_a_* = 10 MPa). Such a difference can be attributed to the presence of the groove in the FEM, which increases the contact surface area between the adhesive and the concrete substrate, leading to enhanced strain transmission compared to the geometrical configuration without groove. 

As a general and important trend, both analytical and numerical approaches show that decreasing the adhesive stiffness lowers the strain transferred to the DOFS core, due to redistribution of the load over a broader effective length. Similar results were reported by Zhou et al. [33]. In addition, the strain transfer is less affected when decreasing the adhesive stiffness for the configuration with groove compared to the two configurations without groove.

**Figure 5:**
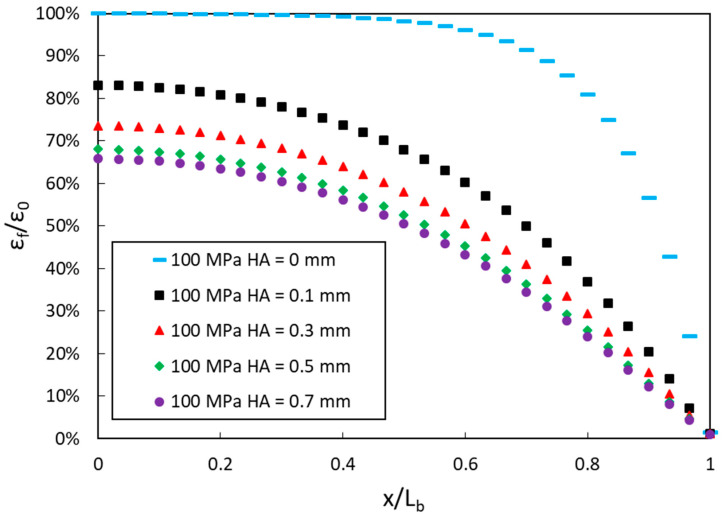
Strain transfer curves provided by the finite element model (FEM) for different values of *HA* (height of adhesive from the bottom of the distributed optical fiber sensor (DOFS) to the concrete surface in the groove).

In a real field instrumentation involving DOFS engraved at the surface of a RC structure, *HA* is usually different from 0 mm (a layer of polymer adhesive lies between the bottom of the DOFS and the concrete surface) and its actual value is very dependent on the initial viscosity of the polymer adhesive during implementation. A complementary FEM simulation was thus conducted to investigate the effect of *HA* on the stress transfer process, considering a fixed value of the adhesive stiffness (100 MPa). Results are displayed in Figure 5 and show that the strain transfer process is negatively affected by an increase in *HA* (similar trend was reported by Ansari et al. [33]). However, this effect decreases considerably when *HA* reaches values over 0.5 mm in the present case.

The dependence of the strain transfer process on the adhesive stiffness, as previously highlighted by theoretical approaches, suggests that it might be beneficial to use a soft polymer adhesive in the framework of crack monitoring applications. Indeed, crack opening generates a strain peak in the host material [7,29], which could be distributed over a broader length of the fiber core when a soft adhesive is used to bond the DOFS to the concrete surface. This solution may reduce the risk of DOFS breakage due to crack development, hence ensuring better durability of the SHM instrumentation under service condition. This concept is explored in the following experimental part.

## 3. Experimental Studies

In this section, two separate experiments are conducted to confirm the influence of the stiffness of the polymer adhesive used to bond/seal the DOFS at the surface of a concrete element on the collected strain profile. These experiments are a compression test on a concrete cylinder and a three-point bending test on a notched concrete prism, which allow respectively performing crack monitoring in the cases of multiple and single crack development.

Different polymer adhesives showing a broad stiffness range were selected for bonding the DOFS to the concrete specimens. Values of their elastic moduli were either provided by the manufacturer or determined experimentally by tensile tests according to EN ISO 527-2 standard [34]. These tests were carried out on series of dumbbell samples at a displacement rate of 1 mm/min, using a universal testing machine (UTM) (Model 5969 from Instron, Norwood, MA, USA) equipped with an advanced video extensometer that measures the differential displacement between two white marks at the surface of the sample. Table 2 provides a list of the various adhesive systems together with their characteristics. It is to note that commercial names or brands of the products are not explicitly reported for confidentiality reasons.

**Figure 6:**
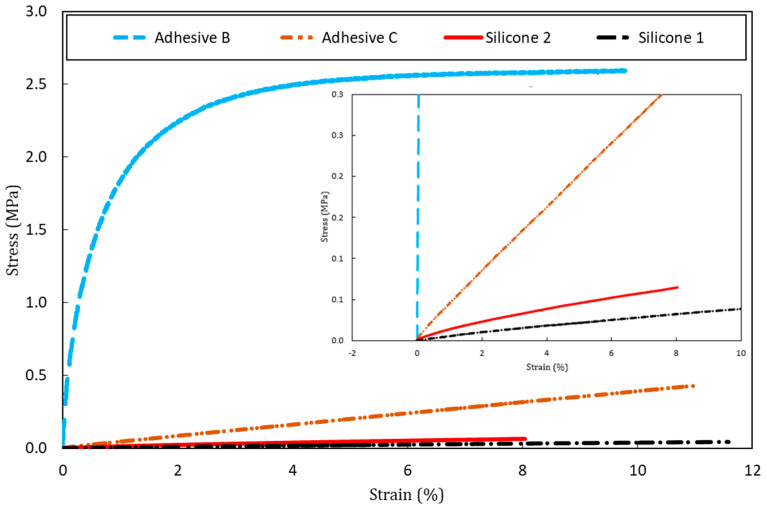
Experimental stress/strain curves collected from samples of the various adhesives subjected to tensile tests.

It is important to mention that, with the exception of Adhesive B, all the adhesives tested showed almost elastic linear behavior (silicones 1 and 2 for strain values up to 8%) or perfectly elastic linear behavior (Adhesive C, even for strain values exceeding 10%) (see Figure 6). This information will be useful to validate the assumptions of the FEM model presented in Section 4.

On the other hand, Adhesive A is brittle with an almost elastic linear behavior up to failure at a strain value 0.4%. The manufacturer’s technical data sheet indicates an average tensile strength of 27.5 MPa.

### 3.1. Compression Test on a Concrete Cylinder

#### 3.1.1. Test Procedure

**Figure 7:**
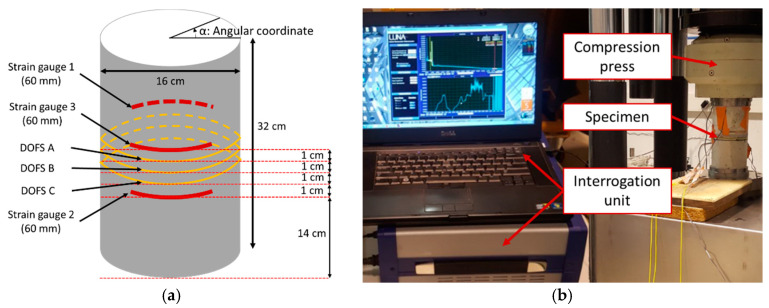
(**a**) Schematic description of the compressive test specimen; (**b**) experimental setup used for the compression test and the interrogation of DOFS.

**Figure 8:**
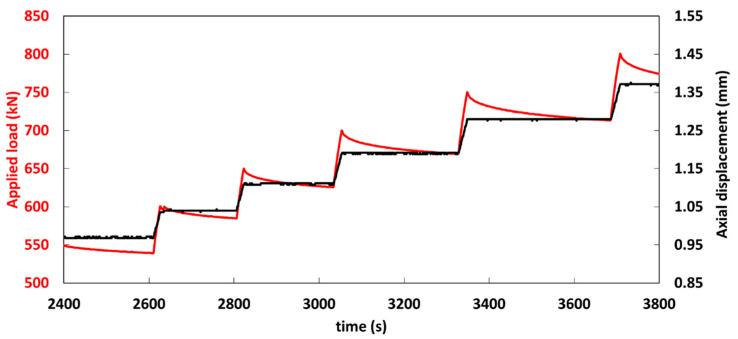
Time evolutions of the applied displacement and corresponding load during the compression test.

A cylindrical concrete specimen (compressive strength of 46 MPa), with a length of 32 cm and a diameter of 16 cm, was used for this test. Three grooves of depth 2 mm and spaced 10 mm apart from each other were carved at the mid-height of the cylinder using a concrete saw. A single mode commercially available DOFS (OF with its primary PI coating of external diameter 160 μm) was sealed in the grooves using three different polymer adhesives (Adhesive A, Adhesive B, and Adhesive C; one type of adhesive per groove), as shown in Figure 7a. The concrete cylinder was then subjected to compression loading up to failure using a hydraulic universal testing machine of capacity 5000 kN (Figure 7b). The test was performed by applying displacement-controlled loading, and the displacement was maintained constant at several preselected loading steps. Such loading protocol was chosen to avoid stability-loss during the crack initiation and propagation processes. Moreover, it enables to keep a constant deformation of the concrete cylinder during maintained load stages (and consequently a constant strain at the location of measurements). The test loading sequence is shown in Figure 8. Strain measurements were collected at the preselected constant displacement levels by interrogating the DOFS with an OBR unit based on optical Rayleigh scattering (OBR-4600 from Luna Technologies) which is shown in Figure 7b. This sensing chain provides the distributed strain profiles along the DOFS with millimetric spatial resolution. Three 60 mm long strain gauges (SGs) were also bonded to the specimen and served as reference instrumentation for comparison with DOFS measurements.

#### 3.1.2. Comparison between DOFS and SG Measurements

In order to compare experimental data provided by DOFS with SG measurements, strain values recorded by each DOFS were averaged over a length of 60 mm covering the same angular coordinates α (see Figure 7a) as those of SGs. The spatial resolution of the interrogation unit was set to 10 mm, and the mean DOFS strain was thus averaged over six collected values.

**Figure 9:**
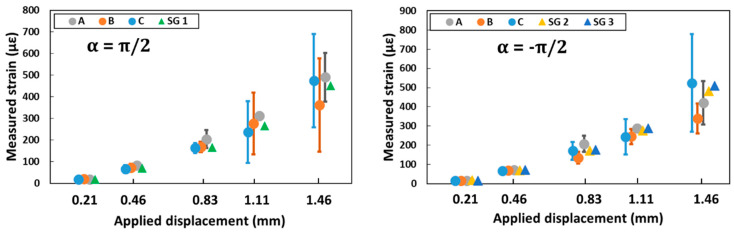
Comparison of strain gauges (SGs) and optical fiber (OF) measurements.

Figure 9 compares strain measurements from SGs at a given displacement level with mean strains (and their standard deviations) provided by DOFS instrumentation. Note that SG 2 and SG 3 are located at the same polar coordinates but at different heights of the concrete cylinder (Figure 7a).

Globally, good agreement is found between the two measurement methods. However, since concrete is a heterogeneous material, large scatter is observed along the 60 mm DOFS length (as shown by the large standard deviations, especially for load levels corresponding to 1.11 mm and 1.46 mm of applied displacement). Such scattering can only be detected using distributed sensors with high spatial resolution, like in the present study. When considering applied displacements below 1.46 mm, it seems that increasing the stiffness of the adhesive leads to an increase in the mean strain values determined from DOFS measurements. This result is probably due to the more significant attenuation of stress transferred to the sensor with the softer adhesives. For an applied displacement of 1.46 mm, the very large standard deviations do not allow to conclude on the influence of the adhesive.

#### 3.1.3. Analysis of Strain Measurements

**Figure 10:**
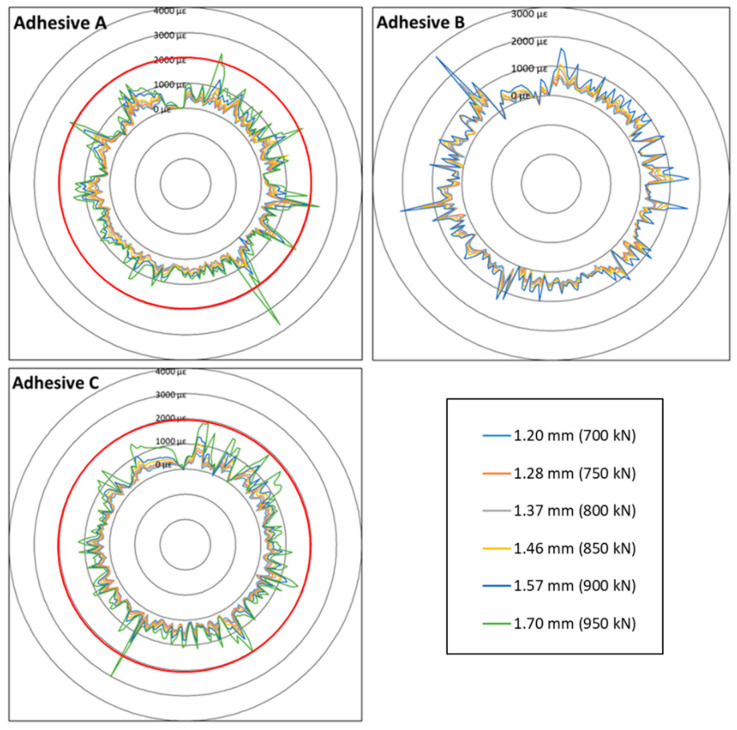
Orthoradial strain profiles around the concrete cylinder, collected at different levels of compression load by DOFS bonded with the three different adhesives (A, B, and C).

Figure 10 illustrates the orthoradial strain profiles (i.e., axial strain along the circumference of the concrete cylinder) recorded by the DOFS bonded with the three different polymer adhesives. Interrogation was carried out at different levels of the applied displacement and the spatial resolution was set to 2 mm in this case. It should be noted that the connector binding the DOFS bonded with Adhesive B to the interrogation unit was slightly damaged. Optical losses were noticed at the first load level and it was impossible to collect data beyond 1.57 mm of applied displacement.

In order to highlight the effect of the adhesive stiffness, one can focus on the comparison of strain profiles obtained with DOFS bonded using systems A and C, which are respectively the most rigid and the softest polymer adhesives among those considered in this study. Although the two corresponding DOFS are not bonded exactly at the same height of the cylinder, several conclusions can be drawn regarding the shape of the strain peaks related to crack development in concrete:

- For a given compression load level, the mean height of the strain peaks increases as the elastic modulus of the adhesive is raised. For example, at the load corresponding to an applied displacement of 1.70 mm, more peaks exceeding 2000 με (this strain level is identified by the red circle in Figure 10) are detected along the DOFS bonded with Adhesive A (6 peaks) compared to the sensor bonded with Adhesive C (only 2 peaks detected).
- In addition, the width at half height of the peaks is much narrower in the case of the stiff polymer adhesive (Adhesive A) compared to softer one (Adhesive C).

These results show that the stiffness of the adhesive has a major influence on the load transfer process near crack locations, as it controls the characteristics of the strain peak that is transmitted from the host concrete structure to the DOFS core. Bonding the DOFS with a soft adhesive allows to distribute stress over a broader length of the sensor and reduce the peak amplitude, compared to using stiff adhesive; hence, mitigating local stress concentrations along the fiber core. In this context, a soft intermediate adhesive layer is expected to ensure better protection of the bonded sensor under usual service condition, i.e., on cracked RC structures.

This first mechanical test allowed a preliminary investigation of the influence of the adhesive properties on the strain response of bonded DOFS instrumentation, by analyzing the shape of strain peaks resulting from multiple crack development in the loaded concrete specimen. These preliminary results, which were also supported by SG measurements, are consistent with trends previously raised from numerical simulations in Section 2. 

### 3.2. Bending Test on a Notched Concrete Prism

#### 3.2.1. Test Procedure

**Figure 11:**
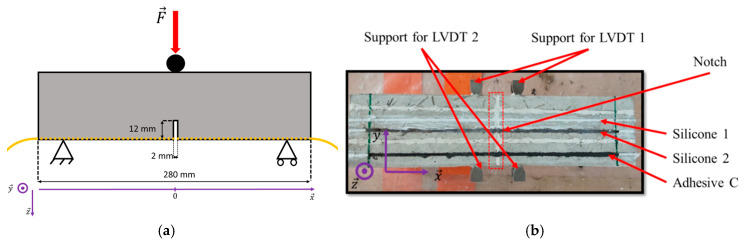
(**a**) Scheme of the bending test configuration; (**b**) bottom view of the prismatic specimen used for the three-point bending test.

A three-point bending test was carried out on a notched prismatic steel fiber-reinforced concrete specimen (dimensions 7 × 7 × 28 cm^3^ and flexural tensile strength of 6.8 MPa) in order to evaluate more precisely the effect of the adhesive stiffness on the strain response of bonded DOFS when a single crack develops in the concrete medium. The size of the notch was scaled proportionally to the dimension of the standard test specimen defined in NF EN 14,651 [35] (Figure 11a). During the test, notch opening was continuously monitored using conventional linear variable differential transformer (LVDT) instrumentation, with micrometer spatial resolution of the overall data acquisition system. LVDT sensors (LVDT 1 and LVDT 2, see Figure 11b) were installed on both sides of the specimen using metallic supports. It must be mentioned that a small height difference may exist between the locations of these LVDTs.

In this experiment, only soft polymer adhesives were used (Adhesive C, Silicone 1, and Silicone 2—see Table 2), as these soft intermediate layers were previously found to reduce stress concentrations along the fiber core. DOFS were sealed in grooves at the concrete surface using the same procedure detailed in Section 3.1.1, and they were interrogated using the same OBR unit as in the previous experiment.

**Figure 12:**
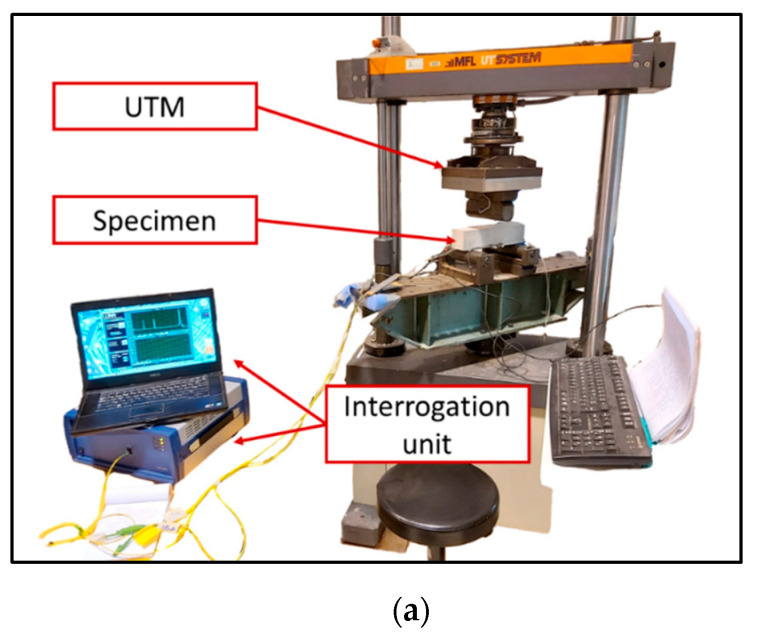

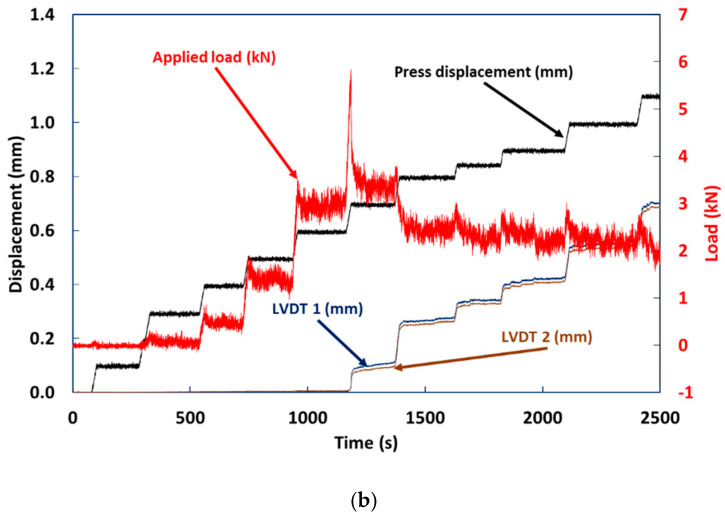
(**a**) Experimental setup used for the bending test; (**b**) recorded time evolutions of the applied load, actuator displacement, and LVDTs displacements.

The bending test was carried out using a Universal Testing Machine of capacity 100 kN (see Figure 12a). Motivated by the same objective as in the previous study (i.e., stability during the cracking process and strain profile remaining constant at a given displacement level), this experiment was conducted under displacement-control loading (Figure 12b). After every increment of 0.1 mm, the machine displacement was held constant for a certain time (as depicted in the loading sequence of Figure 12b) in order to collect DOFS data with the OBR interrogation unit.

#### 3.2.2. Analysis of DOFS Strain Profiles

LVDT signals recorded during the test are presented in Figure 12b. It is found that LVDTs start to detect clearly a notch opening at the sixth plateau corresponding to an UTM actuator displacement of 0.7 mm, which occurs right after the abrupt load drop related to the specimen cracking. The very slight gap between the two LVDT profiles may be related to the previously mentioned possible difference in the heights of locations of the sensors. 

**Figure 13:**
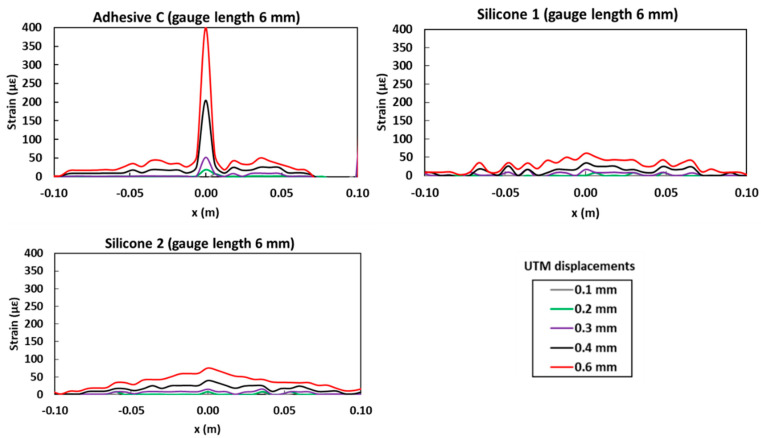
Strain profiles recorded by DOFS bonded to the concrete specimen with three soft adhesives (Adhesive C, Silicone 1, and Silicone 2).

Strain profiles provided by the bonded DOFS for every actuator displacement level up to 0.6 mm, and for the three polymer adhesives used in this experiment (Adhesive C, Silicone 1, and Silicone 2), are illustrated in Figure 13. Unlike LVDT sensors, DOFS were able to detect notch opening at an early stage, much before the sixth plateau (actuator displacement of 0.7 mm). In particular, the DOFS bonded with Adhesive C revealed the development of a strain peak in the central part of the profile (at the abscissa corresponding to the location of the notch) from the second plateau (i.e., actuator displacement of 0.2 mm). DOFS bonded with the two silicone adhesives did not show clear peaks development but rather triangular shaped profiles with a summit located at abscissa *x* = 0 mm (center of the notch), visible from the third displacement level of the UTM actuator (displacement of 0.3 mm). Indeed, the very low stiffness of these silicone adhesives (about 5 times lower than that of Adhesive C) favored the diffusion of stress concentration (induced by notch opening) over the entire length of the bonded DOFS, hence producing this type of triangular strain profile together with drastic reduction of the maximum strain value. 

**Figure 14:**
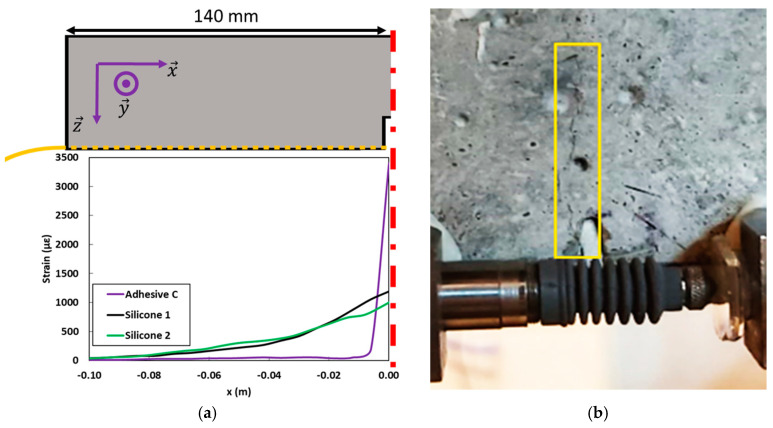
(**a**) Strain profiles along the bonded DOFS at the sixth displacement plateau (over the half concrete prism only); (**b**) aspect of the crack near the initial notch.

Figure 14a shows the strain profiles recorded by the various bonded DOFS for a displacement of the UTM actuator of 0.7 mm (i.e., on the sixth plateau). At this level, strain peaks become clearly visible on all DOFS signals. Nevertheless, the amplitude of this strain peak still remains very dependent on the stiffness of the intermediate adhesive layer, as this latter controls the strain diffusion process from the cracked concrete structure to the DOFS core. In addition, at this displacement level, a crack becomes clearly visible on a lateral side of the specimen, starting from the notch tip and propagating over significant height of the sample (Figure 14b).

In summary, using soft adhesives to seal or bond the DOFS at the surface of the host concrete structure allows to reduce the amplitude of the local strain peak induced by crack opening. Such a mitigation effect seems very dependent on the Young’s modulus of this intermediate adhesive layer. Consequently, the sensor is protected from early breakage while retaining its ability to detect and locate a crack before it becomes visible to the naked eye or detected by conventional sensors. However, in case of very soft adhesives (like silicone systems in the present study), stress concentrations are redistributed over a broad length of the sensing element (sometimes over the entire bonded length), and thus the strain peak is barely detected in the very early stage of investigation, especially in a situation where many cracks appear over a short distance. Therefore, a too soft adhesive may limit the sensitivity of bonded DOFS for crack detection. The adhesive stiffness should thus be optimized to ensure a good compromise between protective function and crack monitoring performance of the bonded DOFS, in relation with the environmental conditions experienced by the host structure. 

## 4. Generalized Strain Transfer FEM Including the Effect of Crack Opening

In this last part of the study, the simplified FEM presented in Section 2 for describing the strain transfer process between the host concrete structure and the bonded DOFS is generalized to take into account crack development. This model is then confronted to experimental evidences from the bending test (Section 3.2) and serves to establish a simple analytical law allowing basic estimation of crack opening in real SHM applications with bonded DOFS.

### 4.1. Representative FEM Geometry

**Figure 15:**
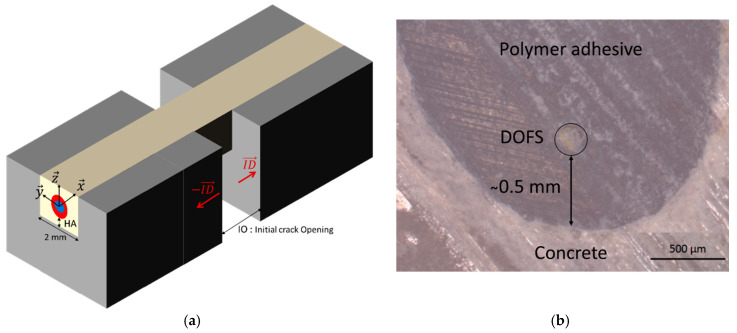
(**a**) Simplified model for the numerical approach of the crack opening; (**b**) actual cross section of an instrumented specimen showing a DOFS sealed in a groove (optical micrograph).

Figure 15a provides a schematic description of the geometry used to build the FEM with crack opening. The model considers the presence of a pre-existing crack (or a notch), and opposite relative displacements ($\vec{ID}$) are imposed to the concrete blocks adjacent to the crack in order to simulate the crack (or the notch) opening. Geometrical parameters that need to be defined in the boundary conditions are the initial crack opening (noted *IO*) and the thickness of adhesive layer between the bottom of the DOFS and the concrete surface (*HA*). Here again, this model was implemented in Cast3m finite element code using linear tetrahedral elements.

### 4.2. Validation of the Model: Simulation of the Bending Test

In order to validate the model, numerical simulations of the bending tests were carried out and compared with experimental results. 

In this generalized model, hypotheses are kept unchanged (Section 2): all materials exhibit linear elastic behaviour and perfect bonding is assumed at interfaces (the validity of such assumption will be discussed in the following). 

Initial boundary conditions were set in accordance with the experimental configuration. The initial crack opening (*IO*) was set to the same value as the experimental notch opening of the concrete specimen, i.e., 2 mm. Furthermore, observations with an optical microscope showed that the actual height of adhesive below the DOFS (*HA*) lies in the range 0.3 to 0.9 mm (an example is shown in Figure 15b). Different values of *HA* in this range were tested numerically and 0.5 mm was found to achieve the best fitting and was thus retained in the numerical simulations.

**Figure 16:**
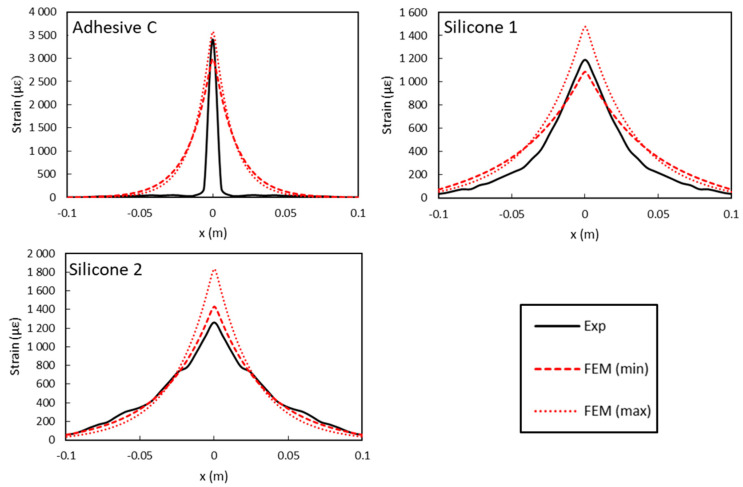
Comparison of numerical simulations (considering extreme values of the adhesive Young’s modulus—min and max) with the experimental (exp) strain profiles (gauge length: 6 mm) collected by DOFS for a displacement of 0.7 mm of the universal testing machine (UTM) actuator.

As experimental elastic moduli of the various adhesives showed relatively large standard deviations (Table 2), numerical simulations were carried out by considering the extreme values of modulus (minimum and maximum) measured for each adhesive. Figure 16 compares the numerical simulations to the experimental strain profiles recorded by the bonded DOFS at a global displacement of 0.7 mm of the UTM actuator during the bending test (sixth displacement plateau), and for the various polymer adhesives (Adhesive C, Silicone 1 and Silicone 2). LVDT measurements were used to provide ($\vec{ID}$) values imposed in the numerical calculations.

**Figure 17:**
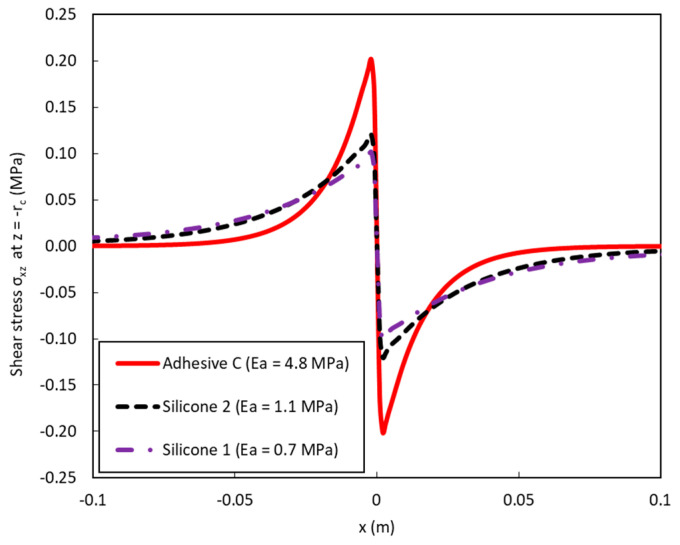
Numerical simulations of shear stress *σ_xz_ (x)* at the adhesive/DOFS interface (*z* = −*r_c_*) considering opposite loads (0.045 MPa) applied to the two concrete blocks adjacent to the crack.

On the overall, numerical results presented in Figure 16 are found in good agreement with experimental data. However, in the case of the DOFS bonded with Adhesive C, the model overestimates to a large extend the width of the strain peak compared to the experiment. Such a difference may result from an experimental sliding effect at the adhesive/DOFS interface on both sides of the notch, which could be significant in the case of Adhesive C and much less pronounced for the very soft silicone systems. Indeed, for opposite loads applied to the concrete blocks adjacent to the crack, the maximum shear stress value *σ_xz_* at the adhesive/DOFS interface (*z* = −*r_c_*) decreases for softer adhesives but spreads over a broader length (see Figure 17).

Anyway, it can be concluded from this part, that the amplitude of the strain peak is globally well predicted by the model, for the three adhesives considered in this experiment. 

In addition, the peak strain in the adhesive layer was also evaluated using the same FEM approach, in order to verify that the polymer remains in the elastic phase during the bending test. It was found that the peak strain in the adhesive close to the DOFS interface (at *x* = 0 and *HA* = 0.5 mm) at the sixth displacement plateau is about 2% for the three soft adhesives used in the bending test. Furthermore, the peak strain drops below this value when moving away from the origin along the $\vec{x}$ axis. Tensile tests have shown previously that the soft adhesives are still in the linear elastic domain at this level of strain (Figure 6). In this context, the assumptions made in the FEM, i.e., linear elastic behavior of materials and interfaces can be considered as relevant. 

### 4.3. Application of the FEM to the Assessment of Crack Opening

The last objective of this study is to apply the proposed numerical approach in view of assessing crack opening from any strain profile provided by a DOFS sealed in a grove at the surface of an existing concrete structure. For this specific geometrical configuration, it was previously shown that the strain transfer process from the host structure to the core OF depends on three main parameters: the elastic modulus of the adhesive, as already highlighted by the results of Figure 16, but also the adhesive height (*HA*), and the initial crack opening (*IO*). In practice, the value of *HA* may vary within a certain range, depending on the experimental protocol used for installing DOFS at the concrete surface, and especially on the initial viscosity of the polymer adhesive. Regarding the initial crack opening *IO*, it can be equal to zero if no crack is present at the moment of the DOFS installation, or it can have a finite value in the case of pre-existing crack (or notched specimen). 

Several analytical models have been proposed in the literature to predict the shape of the strain response of DOFS in the presence of crack. Feng et al. [36] developed a first theoretical approach to describe the strain transfer process in zones containing strain singularities (cracks), in the case of a DOFS sensor bonded to the surface of a host material. Their approach considers the elastoplastic behaviour of the primary coating. Henault et al. defined a mechanical transfer function (MTF) that relates the strain profile along the DOFS core to the strain profile of the host cracked concrete (assuming this latter is a Dirac distribution) [7,21]:

(9) $$MTF\;\left(x\right)=ke^{-k\left|x-x_{c}\right|}$$

where *x* is the abscissa along the DOFS; $x_{c}$ is the crack location (i.e., the abscissa at the summit of the strain peak); and *k* is the “strain-lag parameter” [7,21,37,38,39], which is function of the mechanical and geometrical properties of the different materials (concrete and various components of the DOFS) and the interfaces in-between. 

The same exponential distribution was also presented in the analytical model developed by Imai et al. [40], which adopts the hypothesis proposed by Duck et al. [41]. This latter considers that a discontinuity of the host material (gap or crack) produces a Gaussian strain distribution at the contact interface with the primary coating of the OF. In the same vein, Bassil et al. [42,43] used a similar expression to calculate the shear lag parameter (*k*) of a DOFS cable:

(10) $$\varepsilon \left(x\right)=\frac{COD}{2}ke^{-k\left|x-x_{c}\right|}$$

where *ε(x)* is the strain along the DOFS and *COD* is the crack opening displacement.

According to Equation (10), strain recorded by the DOFS at the crack location $(x=x_{c}$) increases linearly with *COD*. 

**Figure 18:**
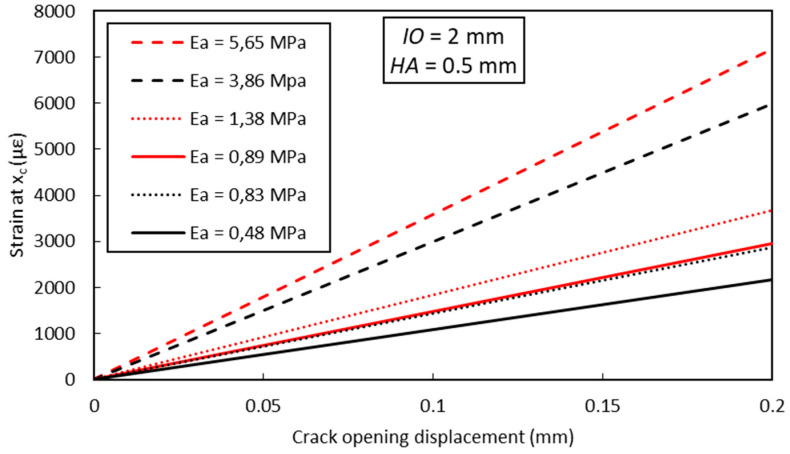
FEM calculation of the peak strain value versus crack opening displacement for different values of the adhesive elastic modulus.

Using the FEM approach proposed in Section 4.1, and considering the same values of parameters *HA* and *IO* previously used to simulate the bending test on the notched concrete prism (*HA* = 0.5 mm and *IO* = 2 mm), the evolution of the peak strain (i.e., strain at the crack location *x_c_*) as a function of the crack opening displacement was predicted. Results are displayed in Figure 18, considering different stiffness values (*E_a_*) of the polymer adhesive used to bond the DOFS. A linear evolution is observed (for a given adhesive stiffness) in accordance with the analytical approach of Bassil et al. [42,43].

**Figure 19:**
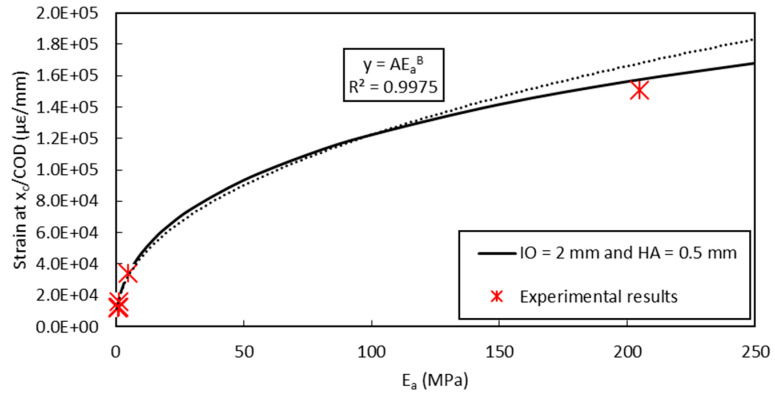
Evolution curve of the shear lag parameter (at *x* = *x_c_*) versus the elastic modulus of the adhesive *E_a_*: FEM simulation (solid line) with exponential regression (dotted line) and experimental results.

Using the same FEM approach, it is possible to predict the evolution curve of the shear-lag parameter (peak strain/*COD*) versus the elastic modulus of the adhesive. FEM simulation and experimental results (obtained from the present bending test at a displacement of 0.7 mm and 0.6 mm of the UTM actuator, and from a complementary bending test involving Adhesive B but not presented in this paper) are illustrated in Figure 19.

Using Equation (10) and results of Figure 19, it is possible to propose an analytical expression of the strain transfer function at the notch level:

(11) $$\varepsilon_{\left(x=x_{c}\right)}=COD.A{\left(E_{a}\right)}^{B}$$

Then, a simple formula can be easily established to assess the crack opening displacement from recorded values of the strain. One must recall that this relationship is only valid in the linear elastic domain of all materials and interfaces:

(12) $$COD=\frac{\varepsilon_{\left(x=x_{c}\right)}}{A{\left(E_{a}\right)}^{B}}$$

Parameters *A* and *B* can be identified from the exponential regression of FEM results in Figure 19. Their values mainly depend on the mechanical and geometrical characteristics of the different material components (with the exception of the adhesive) and on the protocol used to install the DOFS at the surface of the concrete specimen (for instance, sealing in a groove as in this study). 

In order to generalize the analytical expression, the dependence of parameters *A* and *B* to variations in the height of adhesive layer (*HA*) and the initial crack opening (*IO*) must be investigated. In this line, additional FEM simulations were carried out considering changes in the values of *HA* or *IO*, while keeping the other parameters of the model constant. 

**Figure 20:**
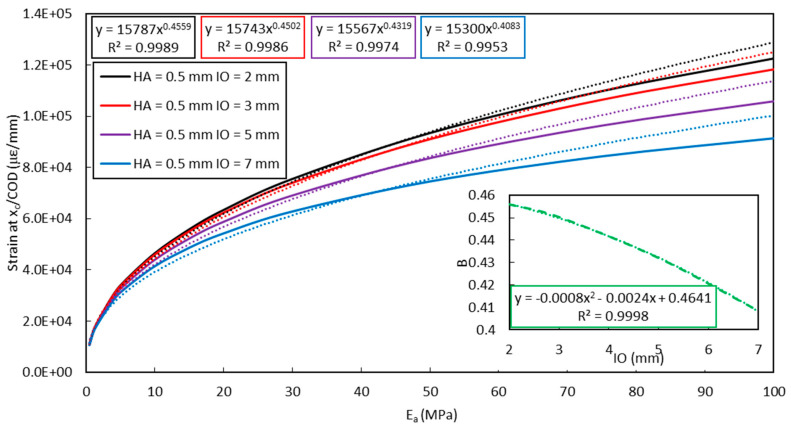
Evolution curves of the shear lag parameter (at *x* = *x_c_*) versus the adhesive Young’s modulus *E_a_*, considering different *IO* values (FEM simulations in solid lines, exponential fitting in dotted lines). Dependence of parameter *B* on *IO* is also displayed as enclosed graph.

Figure 20 shows the evolution of the shear lag parameter (at the notch location, i.e., *x* = *x_c_*) versus the adhesive Young’s modulus *E_a_*, considering different values of *IO*. Values of parameters *A* and *B* are again identified from exponential curve fitting, and it can be noticed that dependence of *A* upon the initial crack opening *IO* is negligible (less than 2% change for an increase in *IO* of 2 mm) while that of *B* remains limited. For *IO* values below 2 mm, one may even assume *A* and *B* almost constant, and the strain transfer process independent of *IO*.

**Figure 21:**
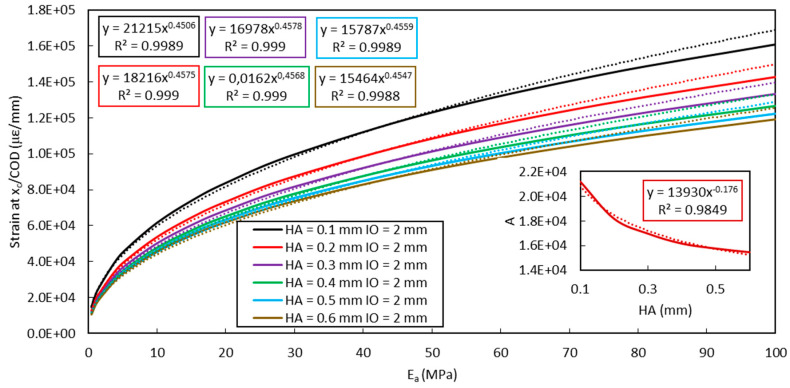
Evolution curves of the shear lag parameter (at *x* = *x_c_*) versus the adhesive Young’s modulus *E_a_*, considering different *HA* values (FEM simulations in solid lines, exponential fitting in dotted lines). Dependence of parameter *A* on *HA* is also displayed as enclosed graph.

In the same way, Figure 21 depicts the effect of *HA* changes on the evolution curves of the shear lag parameter (at *x* = *x_c_*) versus *E_a_*. In this case, numerical simulations show that parameter *A* is an exponential function of *HA* (graph enclosed in Figure 21) and its variation becomes less significant for *HA* values over 0.4 mm. In addition, parameter *B* is a slightly affected by *HA* changes.

In summary, this numerical study served to establish a simplified analytical relationship (Equation (12)) that enables to assess the crack opening from the recorded DOFS peak strain; its validity is restricted to the domain of linear elastic behavior of all material components and interfaces.

This model, developed for the case of DOFS sealed in a groove at the concrete surface using a polymer adhesive, is governed by three physical or geometrical parameters: the initial crack opening *IO*, the height of adhesive *HA* and the elastic modulus of the polymer adhesive *E_a_*. 

When the DOFS instrumentation is installed on a RC structure with no major pre-existing cracks (i.e., initial crack openings below 2 mm, which is the most common case), and the height of the adhesive layer between the DOFS and the concrete surface is large enough (*HA* > 0.4 mm), then coefficient *A* and *B* of Equation (11) can be considered as constants, and the strain response of the bonded DOFS is only governed by the elastic modulus of the adhesive. This configuration is usually met in real case applications.

## 5. Conclusions

In this paper, numerical and experimental investigations are conducted to identify the main parameters governing the strain response provided by DOFS bonded at the surface of an existing RC structure, in view of distributed strain measurement and crack monitoring applications. A special attention is paid to the influence of the stiffness (Young’s modulus) of the polymer adhesive, used to bond or seal the DOFS instrumentation, on the strain transfer process between the host structure and the OF core.

A FEM approach taking into account the actual configuration of the DOFS instrumentation (DOFS sealed in a groove at the surface of the concrete structure) shows that the softer is the adhesive, the lower is the strain transferred to the DOFS glass core due to redistribution over a broader length of the sensor. The same trend was observed experimentally in the framework of a compression test performed on an instrumented concrete cylinder. In the light of these first results, a bending test was then conducted on a notched concrete prism to study the effect of the adhesive stiffness on the strain response of DOFS when a single crack is formed. This test confirmed that the DOFS peak strain induced by crack opening can be drastically decreased when using soft adhesives; hence, preventing early tensile failure of the sensor and potentially extending its service life in the framework of SHM applications on RC structures. However, using too soft adhesives tends to reduce the sensitivity of bonded DOFS instrumentation with regard to early crack detection. The adhesive stiffness should thus be optimized to ensure a compromise between protective function and crack monitoring performance, depending on the in-service environmental conditions of the host structure.

Finally, the FEM approach was extended to the description of the strain transfer process in the case of crack opening. This model allowed to establish a simplified analytical expression that relates the DOFS peak strain to the crack width in the concrete structure. This relationship makes it possible to assess the crack opening from the recorded DOFS peak strain, but its validity is restricted to the domain of linear elasticity of the system. Additional experiments using a broader range of polymer adhesives would be of interest in next studies, to further support the proposed FEM approach and the analytical relationship. The proposed model can be used to analyze recorded strain profile of an instrumented RC structure, or to optimize the adhesive stiffness in function of the expected crack width, in view of ensuring efficient protection of the sensor. 

## Figures and Tables

**Table 1:** Geometrical and mechanical characteristics used in theoretical analysis.

Designation	Notation	Values Used in This Study
Radius of DOFS core	*r_f_*	0.06 mm
External radius of the DOFS	*r_c_*	0.08 mm
Length of the concrete block	*L_b_*	6 mm
Height of host material (concrete) below the DOFS	h	10 mm
Young’s modulus of DOFS core	$E_{f}$	72 GPa
Poisson’s ratio of DOFS core	-	0.17
Young’s modulus of PI coating	$E_{c}$	3 GPa
Poisson’s ratio of the PI coating	-	0.4
Shear modulus of the PI coating	$G_{c}$	1.43 GPa
Young’s modulus of the polymer adhesive	$E_{a}$	Variable (10, 100, and 1000 MPa)
Poisson’s ratio of the polymer adhesive	-	0.48
Shear modulus of the polymer adhesive	$G_{a}$	Variable (depending on $E_{a}$)
Young’s modulus of the host material (concrete)	$E_{h}$	30 GPa
Poisson’s ratio of concrete	-	0.2

**Table 2:** Young’s modulus of the different adhesive systems used in the experimental study.

Designation	Type of Adhesive	Young’s Modulus (MPa)	Standard Deviation (MPa)
**Adhesive A**	Bi-component epoxy system used in construction (highly thixotropic paste)	11,200 ^1^	-
**Adhesive B**	Bi-component epoxy system suitable for bonding variety of materials (high viscosity liquid adhesive)	205	5
**Adhesive C**	Bi-component epoxy system suitable for bonding wide variety of materials (fast cure adhesive)	4.8	1.3
**Silicone 1**	Mastic silicone used for sealing and bonding applications	0.7	0.3
**Silicone 2**	Silicone rubber used to protect strain-gauge installations	1.1	0.4

^1^ Value provided by the manufacturer on the technical data sheet.
